# Hybrid Meat Sausages with Cereal Ingredients: A Systematic Review and Development Trial with the Assessment of Physicochemical and Sensory Attributes

**DOI:** 10.3390/foods13213436

**Published:** 2024-10-28

**Authors:** Anna Olewnik-Mikołajewska, Dominika Guzek, Dominika Głąbska, Krystyna Gutkowska

**Affiliations:** 1 Department of Food Market and Consumer Research, Institute of Human Nutrition Sciences, Warsaw University of Life Sciences (SGGW-WULS), 159C Nowoursynowska Street, 02-776 Warsaw, Polandkrystyna_gutkowska@sggw.edu.pl (K.G.); 2 Department of Dietetics, Institute of Human Nutrition Sciences, Warsaw University of Life Sciences (SGGW-WULS), 159C Nowoursynowska Street, 02-776 Warsaw, Poland; dominika_glabska@sggw.edu.pl

**Keywords:** hybrid meat product, hybrid sausage, semi dry snack sausage, poultry meat, groats

## Abstract

A number of consumers in developed countries are now reducing the amount of meat in their diets, so the development of novel alternatives for conventional meat products is becoming a challenge for the meat industry. The aim of this study was to analyse the possibility of developing hybrid meat sausages with cereal ingredients, based on a systematic review of the literature, as well as a development trial of a hybrid dry snack stick sausage with groats with an assessment of its physicochemical and sensory attributes. A systematic review of peer-reviewed studies about hybrid meat sausages with cereal ingredients, including bibliometric network analysis, was conducted. The development trial was conducted including physicochemical analyses and sensory assessment of the hybrid semi-dry sausages, produced as a ready-to-eat snack (cabanossi) with groats, obtained on an industrial scale. Among the studied hybrid meat products with cereal ingredients, there were patties, frankfurters, salami, and other sausages, while the cereal products added included various components obtained from rice, wheat, chia, and oats. The usefulness of the applied cereal components was emphasised in order to obtain a product of a potential better nutritional value and higher health-promoting properties, as well as being acceptable, or sometimes even better, and described as being products of a good quality. The development trial allowed us to obtain the hybrid semi-dry ready-to-eat cabanossi sausages with groats, which were compared with the hybrid semi-dry ready-to-eat cabanossi sausages with sunflower seeds. Both studied products were characterised by a composition in agreement with requirements, but of a reduced fat content (with hybrid semi-dry ready-to-eat cabanossi sausages with groats being even lower than for those with sunflower seeds; *p* < 0.0001). Their sensory properties were acceptable, even if the plant-based components were recognisable. The characteristics of the hybrid meat products with cereal ingredients both in the literature and development trial were acceptable, and what is even more important is that they are characterised by a potential to be presented as a product of a better nutritional value and higher health-promoting properties.

## 1. Introduction

Meat products, especially red meat, as well as fat meat products and processed meat products, are indicated by the World Health Organisation (WHO) as those that should be reduced in a healthy diet [1]. It is emphasised that health risks resulting from meat consumption increase with the amount of meat consumed, so the reduced consumption of meat is indicated as a major health-related aim [2]. The health risks resulting from meat consumption are associated with numerous problems, as epidemiological studies report that consumption of meat, especially of processed meat and red meat, results in increased mortality, as well as the risk of cardiovascular disease, type 2 diabetes, and certain types of cancer [3]. At the same time, an analysis of the risk of 25 common conditions indicated that increased meat consumption may result in higher risks of ischaemic heart disease, pneumonia, diverticular disease, colon polyps, diabetes, gastro-oesophageal reflux disease, gastritis, duodenitis, and gallbladder disease [4].

Such potential influence of meat results from the fact that its excessive consumption is often associated with overconsumption of energy, resulting in excessive body mass with all its negative consequences [5]. However, the other problem is associated with the composition of meat and meat products, including above all an excessive amount of fat, especially saturated fatty acids [6]. Within the Danish National Survey on Diet and Physical Activity, it was revealed that meat products contributed to 21% of dietary fat, 20% of saturated fatty acids, and 27% of protein [7], also being excessive in a typical diet of developed countries [8] and being associated with health risks [9]. Simultaneously, for plant-based diets, numerous health benefits are indicated, such as reduced risk of type 2 diabetes [10] as well as cardiovascular disease and all-cause mortality [11].

However, health risk associated with increased meat consumption is not the only burden resulting from meat overconsumption, as at the same time, it is indicated that it causes numerous social, environmental, and economic burdens [12]. Taking this into account, the novel trends of reduced meat consumption are becoming popular, including the flexitarian diet, namely, following primarily, but not strictly, a vegetarian diet, with the occasional consumption of meat or fish [13]. At the same time, consumer awareness and motivation for change are increasing in developed countries [14], and currently nearly half of respondents declare following a meat-reduced diet or efforts to reduce the amount of animal products in their diet [15].

Taking this into account, the modern meat consumers have much higher expectations towards meat products than in the past [16]. This results from increased willingness to improve their diet and to reduce meat consumption on the one hand [17], accompanied by some sensory expectations associated with meat products, resulting from hedonism and dependence, on the other hand [18]. As a result, a market of plant-based meat analogues is increasing, but a novel problem arises, as plant-based meat analogues are indicated by the WHO as ultra-processed foods, which means that they may be characterised as having a high energy density, as well as sodium, saturated fats, and free sugars content [19].

Considering the problems described above, development of novel products, being alternatives for conventional meat products, is becoming challenging, as plant-based meat alternatives may be not acceptable for some consumers [20]. Taking this into account, the new category of hybrid meat products has appeared, being based on both meat and plant-based ingredients (but with reduced meat content compared with conventional products) [21], and some attempts have been described to develop hybrid burgers [22,23], sausages [22], and also hybrid meat [24].

The aim of this study was to analyse the possibility of developing hybrid meat sausages with cereal ingredients, based on a systematic review of the literature, and a development trial of a hybrid dry snack stick sausage with groats with the assessment of physicochemical and sensory attributes.

## 2. Materials and Methods

### 2.1. Systematic Review

The literature screening and inclusion of studies were conducted based on the Science Direct and Web of Science databases, and the peer-reviewed studies were included if published up until March 2024.

The studies presenting primary data were allowed to be included, if presenting any study of hybrid meat sausages with cereal ingredients (including both studies based on the technology development and analysis of the final product).

The inclusion criteria were as follows:

(1) research study;
(2) hybrid meat sausages with cereal ingredients studied (any type of minced/homogenised products and any cereal ingredient allowed);
(3) full text of the study published in English;
(4) study published in a peer-reviewed journal.

The exclusion criteria were as follows:

(1) no information about the composition and technology of the studied hybrid meat sausages with cereal ingredients presented;
(2) studies published before 2014 (stated to be out of date).

All the studies that met the inclusion criteria and were not excluded based on the exclusion criteria are presented within the review.

The literature search was based on the Science Direct and Web of Science databases, but in case of review articles, the additional screening of the references was conducted. Two databases were chosen, as a recommended minimum number of databases to improve coverage and recall, as well as to decrease the risk of missing eligible studies [25]. The systematic review was conducted according to the Preferred Reporting Items for Systematic Reviews and Meta-Analyses (PRISMA) guidelines [26]. The electronic searching strategy within the Science Direct and Web of Science databases for hybrid meat sausages with cereal ingredients is presented in Table 1.

**Figure 1:**
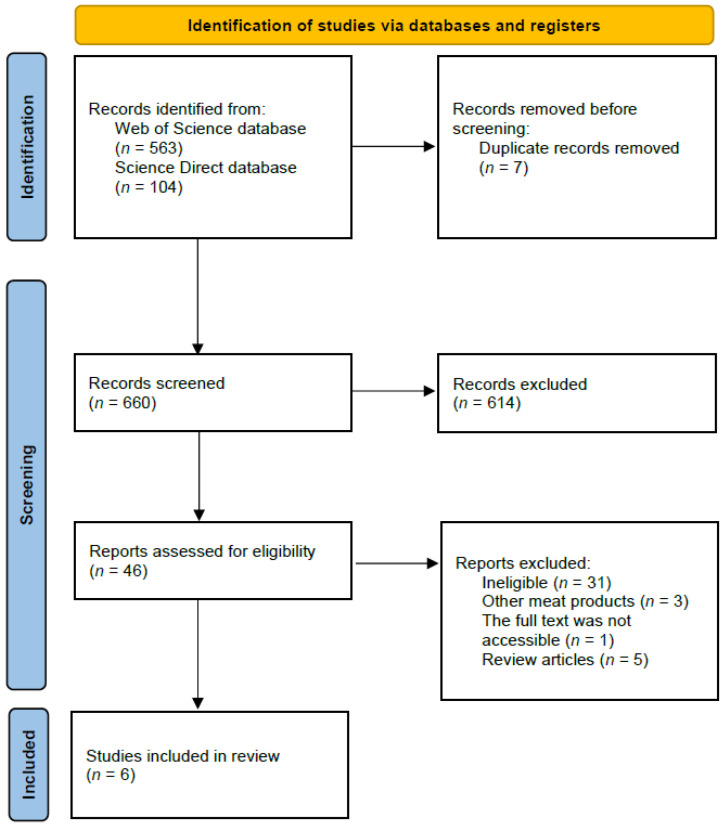
The identification, screening, and inclusion procedure for hybrid meat sausages with cereal ingredients, based on the Science Direct and Web of Science databases.

The identification, screening, and inclusion procedure for hybrid meat sausages with cereal ingredients is reported based on PRISMA guidelines and is presented in Figure 1. The identification, screening, and inclusion were conducted by two researchers independently, while the whole procedure was conducted in 3 stages: based on the title, based on the abstract, and based on the full text of the study. When any disagreement appeared, the third researcher participated in the procedure. After the second stage (assessment based on the abstract), the full texts of the studies were obtained from the electronic databases, but if not available, the corresponding authors of the study were contacted to obtain the full text.

The data extraction procedure was conducted by two independent researchers, based on the full texts of the included studies. When any disagreement appeared, the third researcher participated in the procedure.

The data extracted were organised as follows:

(1) baseline characteristics of the studies included (authors and year of the study, studied meat product, used components, used cereal product);
(2) information about the technology;
(3) observations formulated within the studies included.

Additionally, a graphical analysis was conducted, including the number of scientific publications and bibliometric network analysis.

The number of scientific publications reported by the Science Direct and the Web of Science databases was assessed within the years from 2001 to 2025 (as assessed for September 2024). The analysis of the Science Direct database was conducted for publications containing the term ‘hybrid meat analogs’, and for the Web of Science database, it was conducted for publications containing the term ‘hybrid meat’ (which was associated with the number of publications browsed). Additionally, for the Web of Science database, the bibliometric network analysis of the literature on hybrid meat research using VOSviewer version 1.6.20 (Leiden University, Leiden, The Netherlands) was prepared for the same keywords as within the study for the title and abstract fields ((plant-based meat product OR sausage) AND cereals)). The minimum number of occurrences was chosen as 7, and after choosing 23 terms, 7 redundant terms were removed (type, review, milk, egg, dairy, milk product, study), resulting in the final number of 16 terms within the bibliometric network analysis.

### 2.2. Development Trial

The development trial was conducted within the POIR.01.01.01-00-0524/20 Project by the Polish National Centre for Research and Development, entitled ‘Research and development work on innovative meat and plant products and on elements of proprietary technology for their production, allowing for the market implementation of a new class of convenience food snacks’. As the project aims to develop novel hybrid meat products, some details of the recipe are not possible to reveal until the final product is obtained.

The semi dry sausages, produced as a ready-to-eat snack (in Polish called Kabanosy; in the scientific literature referred to also as cabanossi [27]), were produced with groats, as a plant-based component, to obtain a hybrid meat product.

The studied product was hybrid semi-dry sausages, produced as a ready-to-eat snack (cabanossi) with groats (novel product), while their characteristics were compared with the other hybrid semi-dry sausages, produced as a ready-to-eat snack (cabanossi as well) with sunflower seeds (product already produced). The reference product was chosen, as it was as similar as possible to the novel developed product. Moreover, as hybrid meat products are characterised by reduced meat content, by adding plant-based ingredients [21], various groups of plant-based ingredients are allowed, and the sunflower seed component is a well-known option [28].

The studied products were prepared with the ingredients as follows:

(1) Hybrid semi-dry ready-to-eat cabanossi sausages with groats: chicken meat (70 g per 100 g of product), barley groats (15 per 100 g of product), golden linseed (7 g per 100 g of product), dried apples, salt, rapeseed oil, dried vegetables (tomatoes, carrots, celery, onion, parsnip, leek, pepper, parsley), flavours (including celery, smoke flavour), beetroot, spice extracts (including pepper extract), spices, natural flavours, lovage, garlic, preservative (sodium nitrite).
(2) Hybrid semi-dry ready-to-eat cabanossi sausages with sunflower seeds: chicken meat (75 g per 100 g of product), shelled sunflower seeds (15 g per 100 g of product), golden linseed (7 g per 100 g of product), dried apples, salt, white mulberry, spice extracts (including pepper extract), flavours (including celery, smoke flavour), spices, garlic, natural flavours, preservative (sodium nitrite).

Both products were prepared in an edible casing, namely, sheep intestine, based on the standard procedure, while the major innovation for both products was the reduced meat content compared with the standard cabanossi recipe [29]. The share of animal-based and plant-based ingredients was as follows:

(1) Hybrid semi-dry ready-to-eat cabanossi sausages with groats: animal-based components: 58.95%, plant-based components: 34.53%, other: 6.52%.
(2) Hybrid semi-dry ready-to-eat cabanossi sausages with sunflower seeds: animal-based components: 56.07%, plant-based components: 40.50%, other: 3.43%.

The energy value of the hybrid semi-dry ready-to-eat cabanossi sausages with groats and the hybrid semi-dry ready-to-eat cabanossi sausages with sunflower seeds was calculated according to Regulation (EU) No. 1169/2011 of the European Parliament and of the Council [30] based on the assessed content of the nutrients, and it was expressed in kcal/100 g.

The water content of the hybrid semi-dry ready-to-eat cabanossi sausages with groats and the hybrid semi-dry ready-to-eat cabanossi sausages with sunflower seeds was established using a standard gravimetric method according to PN ISO 1442:2000 [31], and it allowed for the calculation of dry matter (difference of the mass of sample and the water content).

The ash content of the hybrid semi-dry ready-to-eat cabanossi sausages with groats and the hybrid semi-dry ready-to-eat cabanossi sausages with sunflower seeds was established using a standard gravimetric method according to PN ISO 936:2000 [32].

The protein content of the hybrid semi-dry ready-to-eat cabanossi sausages with groats and the hybrid semi-dry ready-to-eat cabanossi sausages with sunflower seeds was estimated by multiplying the determined nitrogen content by a nitrogen-to-protein conversion factor—6.25. The analysis was conducted using a titrimetric method according to ISO 1871:2009 [33].

The fat content after hydrolysis of the hybrid semi-dry ready-to-eat cabanossi sausages with groats and the hybrid semi-dry ready-to-eat cabanossi sausages with sunflower seeds was established using a gravimetric method according to industry standards. The method for analysing ‘fat’ means total lipids, which includes phospholipids, and it complies with Annex I of Regulation (EU) No. 1169/2011 of the European Parliament and of the Council [30].

The fatty acid composition of the hybrid semi-dry ready-to-eat cabanossi sausages with groats and the hybrid semi-dry ready-to-eat cabanossi sausages with sunflower seeds was established using a gas chromatography with flame ionisation detection method according to PN EN ISO 12966-1:2015-01 + AC:2015-06 [34]. The content of saturated fatty acids was presented afterwards.

The fibre content of the hybrid semi-dry ready-to-eat cabanossi sausages with groats and the hybrid semi-dry ready-to-eat cabanossi sausages with sunflower seeds was determined using the Megazyme K-TDFR reagent test, which combines enzymatic and gravimetric methods according to AOAC 991.43:1994 [35].

The sodium content of the hybrid semi-dry ready-to-eat cabanossi sausages with groats and the hybrid semi-dry ready-to-eat cabanossi sausages with sunflower seeds was determined using the flame photometry method according to industry standards. The method for sodium means the salt equivalent content calculated using the formula: salt = sodium × 2.5, and it complies with Annex I of Regulation (EU) No. 1169/2011 of the European Parliament and of the Council [30].

All the analyses were performed in an accredited laboratory (Accreditation certificate of testing laboratory no. AB 1334 by the Polish Center for Accreditation), with accreditation for the used methods.

The sensory analysis was conducted by the GBA POLSKA LLC Sensory Research Laboratory, meeting the requirements of the PN-ISO 8589:2010 standard [36], which guarantees standardised analysis conditions, minimising the impact on test application. It is equipped in independent stations for assessing food products. Sensory analyses were performed by a properly trained team of at least 8 panellists with many years of experience in the assessment of food products. The GBA POLSKA LLC Sensory Research Laboratory has appropriate accreditation in Poland (AB 1334). Descriptive tests were conducted according to the following descriptors: (1) appearance, (2) appearance at the cross-section, (3) aroma, (4) taste, and (5) structure and consistency, and the assessments were conducted after (1) 28 days (4 weeks) of storing the sample at 25 °C; (2) 60 days (2 months) of storing the sample at 25 °C; and (3) 120 days (4 months) of storing the sample at 25 °C. The sensory panel evaluated samples one by one in a random order.

### 2.3. Statistical Analysis

To verify the normality of distribution, the Shapiro–Wilk W test was applied. Differences between the features were assessed using Student’s *t*-test (in the case of normal distribution) or the Mann–Whitney U test (in the case of distribution different than normal). Level of significance *p* ≤ 0.05 was considered statistically significant. Statistical analysis was conducted using Statistica 13.3 (StatSoft, Tulsa, OK, USA).

## 3. Results

### 3.1. Systematic Review

**Figure 2:**
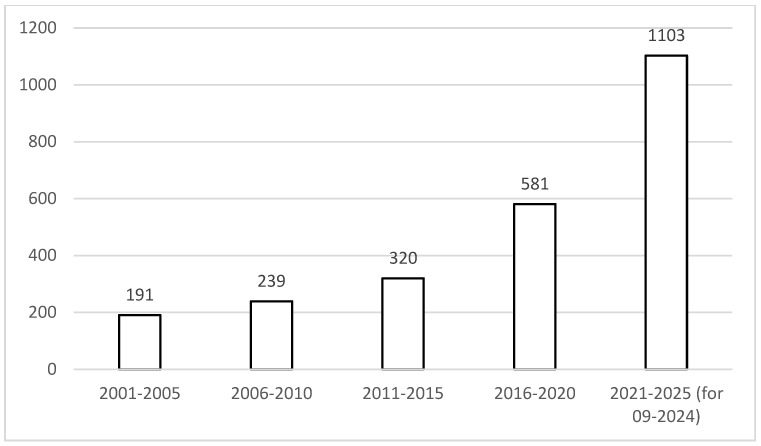
The number of scientific publications reported by the Science Direct database containing the term ‘hybrid meat analogs’ within the years from 2001 to 2025 (as assessed for September 2024).

**Figure 3:**
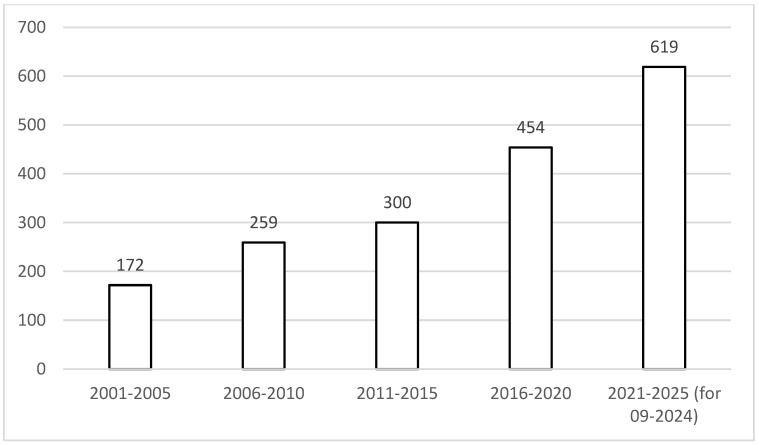
The number of scientific publications reported by the Web of Science database containing the term ‘hybrid meat’ within the years from 2001 to 2025 (as assessed for September 2024).

It may be noticed that the number of scientific publications about the hybrid meat products is constantly growing, being observed both within the Science Direct (Figure 2) and Web of Science databases (Figure 3). Especially for the Science Direct database, it may be indicated that the number of related publications recently doubled each 5 years. At the same time, for the Web of Science database, the growth of the number of publications was less profound, with the number of publications doubling each 10 years.

**Figure 4:**
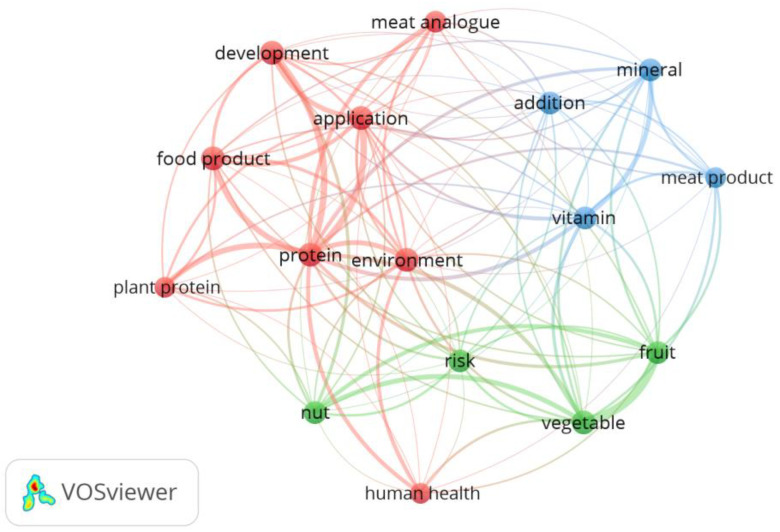
The bibliometric network analysis of the literature on hybrid meat research using VOSviewer (Leiden University, Leiden, The Netherlands) for the Web of Science database.

The bibliometric network analysis of the literature on hybrid meat research using VOSviewer is presented in Figure 4. It was observed that the VOSviewer analysis revealed three major clusters, which may be interpreted as follows: (1) red—associated with the reason for producing hybrid meat products (human health, environment, protein, plant protein, meat analogue) and their production (development, application, food product), (2) the used ingredients and associated risk (nut, fruit, vegetable, risk), and (3) the nutritional value of the final hybrid meat product (vitamin, mineral, addition, meat product).

The baseline characteristics of the studies presenting hybrid meat products with cereal ingredients [37,38,39,40,41,42], included in a systematic review (authors and year of the study, studied meat product, used components, and used cereal product), are presented in Table 2. A relatively small number of studies of hybrid meat products with cereal ingredients were found, wherein they presented a specific product with the composition and technology information. Regarding the agreement with the previously presented number of scientific publications, they were published recently, with one study published in the year span of 2010–2015, two studies published in the year span of 2016–2020, and three studies published since 2021. Among the studied hybrid meat products, there were patties [37,42], frankfurters [38,39], salami [41], and other sausages [40]. The components used were quite typical for the type of product, while the cereal products added included various components obtained from rice [37,41,42], wheat [38], chia [39], and oats [40].

The baseline information about the technology within the studies presenting hybrid meat products with cereal ingredients, included in a systematic review, is presented in Table 3. The applied technology was quite typical for the specific type of product, including patties [37,42], frankfurters [38,39], salami [41], and other sausages [40]. The additional stage of the production was associated with adding plant components in order to obtain a hybrid meat product, while various components obtained from rice [37,41,42], wheat [38], chia [39], and oats were added [40]. They were added depending on the type of meat product and on the added ingredient in the various stages of production—mainly in the final stage before blending and shaping, but sometimes additional preparing of the cereal components was necessary, including its soaking in water [42] or boiling [38].

The observations formulated within the studies presenting hybrid meat products with cereal ingredients included in a systematic review are presented in Table 4. Due to the small number of the studies of hybrid meat products with cereal ingredients having been published so far, the observations formulated within them were associated with various areas. Mainly, the usefulness of the applied cereal components was emphasised, in order to obtain a product of a potential better nutritional value and higher health-promoting properties [37,40,41]. For the physicochemical parameters, it was indicated that the hybrid meat products with cereal ingredients may be acceptable [39], or comparable with typical meat products with no such ingredients [42], or sometimes even better [37], as well as hybrid meat products were described as products of a good quality [40]. The stability depended on the product and was described as either better (which was associated with better resistance to oxidation and lower residual nitrite levels) [39] or worse (which was associated with increases in the total viable count and lactic acid bacteria) [38].

### 3.2. Development Trial

The physicochemical characteristics of the hybrid semi-dry ready-to-eat cabanossi sausages with groats compared with the hybrid semi-dry ready-to-eat cabanossi sausages with sunflower seeds are presented in Table 5. Statistically significant differences between physicochemical characteristics were stated, as the hybrid semi-dry ready-to-eat cabanossi sausages with groats were characterised by a lower energy value (*p* < 0.0001), dry matter (*p* < 0.0001), and fat content (*p* < 0.0001), as well as higher ash (*p* = 0.0356) and sodium contents (*p* = 0.0189).

The sensory descriptors of appearance and consistency of the hybrid semi-dry ready-to-eat cabanossi sausages with groats compared with the hybrid semi-dry ready-to-eat cabanossi sausages with sunflower seeds after storage at 25 °C are presented in Table 6. It may be indicated that for all the samples, the appearance, appearance at the cross-section, and structure and consistency were at least acceptable. Due to the fact that both products were hybrid meat products, the highest notes were formulated for appearance, described as characteristic/quite characteristic, independently from type of hybrid meat products assessed. Interestingly, for appearance at the cross-section, as well as structure and consistency, the hybrid semi-dry ready-to-eat cabanossi sausages with groats and those with sunflower seeds had similar notes as well, but not as good, as formulated for appearance, which probably resulted from visible groats and sunflower seeds, causing the appearance at cross-section and structure to not be so characteristic as expected, being described often as proper/quite proper or acceptable.

The sensory descriptors of aroma and taste of the hybrid semi-dry ready-to-eat cabanossi sausages with groats compared with the hybrid semi-dry ready-to-eat cabanossi sausages with sunflower seeds after storage at 25 °C are presented in Table 7. It may be indicated that for all the samples, the aroma and taste are characteristic/quite characteristic. Interestingly, both for aroma and taste, the hybrid semi-dry ready-to-eat cabanossi sausages with groats after a shorter time of storage (28–60 days) were assessed as quite characteristic, but after longer storage (120 days), they were assessed as characteristic.

## 4. Discussion

Cabanossi is a type of cured and cold smoked sausage stick consumed as a ready-to-eat snack [29]. Cabanossi, under the name of ‘kabanos’, is a Polish traditional meat product that since 2011 has been registered in the European Union as a product of traditional specialties guaranteed (TSG) [43]. According to the Commission Implementing Regulation (EU) No. 1044/2011 of 19 October 2011 entering a name in the register of the traditional specialities guaranteed [43], ‘kabanosy’ are long, thin sticks of dry sausage twisted off at one end and evenly wrinkled, being folded in two, with its surface dark red in colour with a cherry tint; cross-section with dark red pieces of meat and cream-coloured fat; having a smooth, dry, and evenly wrinkled surface; and with a strong taste of cured, baked pork and a delicate, smoky aftertaste redolent of caraway and pepper.

The Commission Implementing Regulation (EU) No. 1044/2011 of 19 October 2011 entering a name in the register of the traditional specialties guaranteed [43] presents also the required composition of ‘kabanosy’, including protein content of not less than 15.0%, water content of not more than 60.0%, fat content of not more than 35.0%, and salt content of not more than 3.5%. All the listed criteria are met in the case of both studied hybrid meat products—the hybrid semi-dry ready-to-eat cabanossi sausages with groats and the hybrid semi-dry ready-to-eat cabanossi sausages with sunflower seeds.

Moreover, it should be indicated that due to plant-based components, it was possible to obtain within a development trial products of a reduced fat content—not only lower than 35%, but for cabanossi sausages with sunflower seeds about 15%, and for cabanossi sausages with groats about 10%. In general, dry and semi-dry sausages contain quite high levels of fat [44], which is now not acceptable by many consumers, while low-fat meat products may satisfy the need of the modern-day health-conscious consumers [27]. Although such modification may improve the nutritional characteristics, simultaneously it could also influence moisture, colour, and texture, as fat in meat products affects the sensory properties and plays a major role in the creation of a desirable appearance, deliciousness, texture acceptability, and a feeling of satiety [45]. Therefore, all changes related to reduced level of fat must be designed not only to improve the nutritional characteristics but also to maintain consumer acceptance of the product. Such a situation was observed for the studied hybrid meat products, as both products were described as at least acceptable, even if the composition was changed and characteristics was not typical (which was stated mainly for appearance at the cross-section, as well as in terms of structure and consistency).

The consumers’ acceptance of a meat product depends on many aspects, including animal welfare; the environmental impact; and their attitudes and beliefs regarding the products, nutrients, or ingredients [46]. Meat alternatives or hybrid meat products with plant-based ingredients may have some advantage, taking into account the so-called ‘health halo’ effect. The ‘health halo’ effect refers to automatic processing of information associated with overestimating the healthfulness of an item based on a single claim [47]. Taking into account that consumers simply classify food products as healthy or unhealthy [48], as well as considering the studies which have shown that food labels influence this classification through the ‘health halo’ effect [49], hybrid meat products can have such an advantage. The positive ‘health halo’ effect was proven in the study of Schösler et al. [50], as even simple information about a product could influence consumers’ caloric estimation, nutritional image (including estimation of fibre content), and even palatability.

The comparison between hybrid meat products and traditional meat products highlights the potential for hybrid options to provide improved nutritional profiles through reduced saturated fat, reduced energy, and increased fibre [51]. Moreover, some research indicates that consumers tend to perceive the nutritional value of such a product more positively [52]. This makes them an appealing choice for consumers looking to maintain meat flavours while increasing their intake of plant-based nutrients. Additionally, hybrid meat products incorporate dietary fibre or antioxidant compounds, which contributes to promoting a more sustainable lifestyle and a functional diet [53]. These products not only may provide essential nutrients and dietary fibre but can also support the prevention of chronic diseases [54].

Bearing this in mind, hybrid meat products as a combination of meat and plant-based ingredients are products dedicated for consumers who want to reduce their meat intake but without losing the taste of meat products [55] and consumers who want to consume products more familiar to them than plant-based alternatives to meat [27], so they can have a competitive advantage on the market. However, it should be mentioned that through the ‘health halo’ effect, such products may cause consumers’ misjudgement and as a consequence lead to an overconsumption of these food products [56].

Nevertheless, the modern food industry is focused on the supply of so-called ‘healthier’ meat products mostly to generate profits in competitive markets [7]. It is associated with a growing amount of research on hybrid meat products, as indicated within the systematic review, in that the number of publications doubled each 5 or 10 years. Such a growing number of studies is associated with growing market needs, as a number of studies are focused on the development trials of various hybrid meat products with cereal ingredients, including patties [37,42], frankfurters [38,39], salami [41], and other sausages [40].

The characteristics of the studied hybrid meat products with cereal ingredients is in general acceptable, and what is even more important is that they are characterised by a potential to be presented as a product of a better nutritional value and having higher health-promoting properties [37,40,41], which is associated with the above-mentioned ‘health halo’ effect.

Taking into account that novel strategies for the meat market are needed in order to overcome not only the health-related problems but also those from the perspectives of sustainability and the economy [8], the hybrid meat products with cereal ingredients may be an interesting option meeting the needs of both producers and consumers.

## 5. Conclusions

The characteristics of the hybrid meat products with cereal ingredients both in the literature and development trials are acceptable, and what is even more important, they are characterised by a potential to be presented as a product of a better nutritional value and higher health-promoting properties.

## Figures and Tables

**Table 1:** The electronic searching strategy within the Science Direct and Web of Science databases for hybrid meat sausages with cereal ingredients.

Database	The Detailed Strategy of the Electronic Searching
Science Direct	Title, abstract, keywords: plant-based meat product AND sausage AND cereals
Web of Science	ALL = ((plant-based meat product OR sausage) AND cereals)

**Table 2:** The baseline characteristics of the studies presenting hybrid meat products with cereal ingredients, included in a systematic review.

Ref.	Author, Year	Studied Meat Product	Used Components	Used Cereal Product
[37]	Gao et al., 2014	Ground pork patties	Fresh pork legs, frozen pork backfat, glutinous rice flour, soy protein isolates, corn starch, potato starch	Glutinous rice flour
[38]	Ranucci et al., 2018	Frankfurters	Lean pork thighs, salt, black pepper, boiled whole emmer wheat, peeled roasted almonds, fermented fish sauce	Emmer wheat (*Triticum dicoccum* Schübler) of protected geographic indication ‘Farro di Monteleone di Spoleto’
[39]	Fernández-López et al., 2019	Frankfurters	Pork lean meat, pork backfat, water, potato starch, sodium chloride, sodium tripolyphosphate, sodium nitrite, casein, liquid smoke and spices (white pepper, mace, coriander)	Chia seeds, chia flour, chia coproduct (milled residue obtained after cold pressing and oil separation from the chia seeds)
[40]	Gore et al., 2022	Minced sausages prepared from Indian major carp	Rohu fish, cod liver oil, corn starch, dietary oat fibre, salt (NaCl)	Oat fibre
[41]	Espinales et al., 2023	Fermented salami	Lean ground pork 90/10 (6 mm coarse), refined salt (NaCl), refined cane sugar, nitrate (curing agent), sodium erythorbate, polyphosphate, probiotic starter culture (*Pediococcus acidilactici* and *Pediococcus pentosaceu*) (LHP), cooked rice	Ungerminated and germinated white rice and brown rice
[42]	Janardhanan et al., 2023	Patties	Veal meat (Biceps femoris), Legumbreta Fina	Legumbreta Fina, a commercially available extruded product made from mixed flours (soy, rice, and bean)

**Table 3:** The baseline information about the technology within the studies presenting hybrid meat products with cereal ingredients, included in a systematic review.

Ref.	Information About Technology
[37]	Deboned pork leg was trimmed to remove connective tissue and visible fat by hand and was stored at 4 °C for 1 d after slaughter for aging; meat and backfat were coarsely minced in a mincer; condiments were added (salt, sodium tripolyphosphate, ice water, nutmeg, sugar, black pepper, onion powder, monosodium glutamate, soy sauce, and rice wine) and were mixed in a laboratory chopper for 2 min at speed 1; glutinous rice flour was added and the mixture was blended; weighed patty mixture was shaped by hand into a meat patty with a diameter of 80 mm and a height of 15 mm; pork patties were placed on an oven tray and cooked in a preheated commercial kitchen oven at 190 °C for 10 min to achieve an internal end-point temperature of 75 °C; patties were cooled to room temperature, vacuum-packaged, and stored at 4 °C.
[38]	Lean pork thighs were deboned, cut in blocks, and ground in a mincer (almost 2 × 2 mm); the mince was mixed with salt, black pepper powder, boiled whole emmer wheat, peeled roasted almonds pieces, and fermented fish sauce and mixed for 5 min and filled through a piston stuffer in pigs’ small intestine casing in order to obtain frankfurters of 50 g with 10 cm length and 1 cm diameter; the frankfurters were oven-cooked until reaching a core temperature of 72 °C for 1 min, cooled down to 2 °C in a chiller, sealed under vacuum, and stored under refrigeration (4 ± 1 °C).
[39]	Meat ingredients were ground in a cutter and mixed with sodium chloride and the rest of the ingredients for 2 min (temperature below 12 °C); after homogenisation, the resulting meat batter was stuffed into 20 mm diameter cellulose casings, hand linked, cooked in a water bath (90 °C) to an internal temperature of 72 °C, immediately chilled in ice for 5 min, peeled by hand, vacuum packed in plastic bags, and stored immediately after packing at 4 °C (±1 °C) under darkness conditions.
[40]	Fishes were filleted, cleaned, and deboned; mince was prepared (drum sieve with 5 mm diameter holes); grinding took place with 2.5% (*w*/*w*) NaCl for 3 min (silent cutter); the chilled mince was comminuted (stainless steel bowl chopper) for 3 min with a slow addition of cod liver oil (8% *v*/*w*); mixing occurred for 3 min, with corn starch 8% (*w*/*w*) added, followed by mixing for 3 min, with oat fibre (2.5%, 5%, 7.5% and 10%) (*w*/*w*) then added, followed by further mixing for 3 min; the mixture was filled manually (hand stuffer) into a krehalon casing of 2.5 cm diameter; the ends of the tubes were tied; pre-incubation took place at 40 °C for 30 min, followed by heating in a water bath at 90 °C for 20 min.
[41]	Salami formulation was stuffed into a natural casing (manual plastic stuffer of 22 mm diameter); 24 h fermentation-drying at 32 ± 1 °C and 85% relative humidity (incubator) was applied until the pH was below 5.1; ripening (refrigerator) occurred at 5 °C and 80% relative humidity for 25 days.
[42]	Legumbreta Fina was soaked in water; external connective tissue and visible fat of the meat were trimmed off; meat was reduced to cubes and minced at low speed for 20–30 s at 20 °C using a meat grinder machine, and patties were prepared by mixing meat and plant-based product, formed into the patties’ shape between two grease-proof paper sheets using a patty press; samples were vacuum packaged (98% vacuum) in bags and stored at 4 °C for 24 h.

**Table 4:** The observations formulated within the studies presenting hybrid meat products with cereal ingredients, included in a systematic review.

Ref	Conclusion
[37]	Glutinous rice flour patties were juicier, more tender, and showed better flavour and overall acceptability compared to the control and the treatments with other additives. The results indicate that glutinous rice flour could be an effective functional ingredient in ground pork patties.
[38]	During the 24 days of storage, the product evidenced a decrease in the pH and increases in the total volatile nitrogen content and TBARs value, whereas the aw remained stable. From a microbiological perspective, increases in the total viable count and lactic acid bacteria up to 4.8 log cfu/g occurred during storage, but no pathogens were found. Sensory analyses revealed a change in odour and flavour at 18 days, with the detection of a fermented and rancid taste. Survival sensory analysis defined a shelf life of the products of between 18.6 and 22.7 days.
[39]	Although differences were detected in the sensory attributes of the frankfurters reformulated with chia products (most of them when chia coproduct was added), all of them were judged as acceptable. Besides the quality aspects, these reformulation strategies had beneficial effects on some technological properties during chilled storage: better resistance to oxidation (controlling the TBARs increase during storage) and lower residual nitrite levels than the control (both effects presumably because of the chia polyphenol content) and no effect on microbiological safety.
[40]	Fortification of minced sausages with oat fibre at the level of 2.5% (*w*/*w*) is recommended. The fortification of minced sausages with fibre containing starch and cod liver oil resulted in a good-quality product that could be useful in developing mince-based fish products with nutritional benefits.
[41]	The addition of cooked rice improved the nutritional composition of salamis by decreasing fat and increasing total and insoluble fibre content and enhanced their health-promoting properties due to the increase in bioactive compounds and antioxidant activity.
[42]	The effect of high-hydrostatic pressure processing on hybrid patties was not comparable to veal patties. The dual (high-hydrostatic pressure processing and sous-vide cooking) technology has the potential to develop novel hybrid products with physicochemical characteristics comparable to those of veal-based patties.

**Table 5:** The physicochemical characteristics of the hybrid semi-dry ready-to-eat cabanossi sausages with groats compared with the hybrid semi-dry ready-to-eat cabanossi sausages with sunflower seeds.

Nutritional Value (per 100 g of Product)	Hybrid Semi-Dry Ready-to-Eat Cabanossi Sausages with Groats		Hybrid Semi-Dry Ready-to-Eat Cabanossi Sausages with Sunflower Seeds		*p*
	Mean ± SD	Median (25th–75th)	Mean ± SD	Median (25th–75th)	
Energy value (kcal)	241.18 ± 7.19	242 (11)	309.67 ± 13.5	310 (27)	<0.0001
Dry matter (g)	56.77 ± 1.43	56.5 (2.7)	65.07 ± 3.18	66.7 (5.7)	<0.0001
Ash (g)	4.89 ± 0.35	4.95 * (0.17)	4.22 ± 0.09	4.18 (0.17)	0.0356
Protein (g)	24.16 ± 1.98	24.75 * (2.12)	24.21 ± 0.91	24.19 (1.82)	0.6971
Fat (g)	9.81 ± 1.11	10.23 (1.51)	15.24 ± 1.95	14.94 (3.87)	<0.0001
Saturated fats (g)	2.74 ± 0.29	2.78 (0.37)	3.14 ± 0.97	2.71 (1.8)	0.2309
Fibre (g)	7.76 ± 2.85	7.5 (4.2)	5.0 ± 1.47	4.2 (2.6)	0.1383
Sodium (g)	1.36 ± 0.11	1.39 * (0.09)	1.04 ± 0.02	1.04 (0.03)	0.0189

* nonparametric distribution (verified with Shapiro-Wilk W test; *p* < 0.05)

**Table 6:** The sensory descriptors of appearance and consistency of the hybrid semi-dry ready-to-eat cabanossi sausages with groats compared with the hybrid semi-dry ready-to-eat cabanossi sausages with sunflower seeds after storage at 25 °C.

Descriptors	Time of Storage	Hybrid Semi-Dry Ready-to-Eat Cabanossi Sausages with Groats	Hybrid Semi-Dry Ready-to-Eat Cabanossi Sausages with Sunflower Seeds
Appearance	28 days	quite characteristic for this product, edible casing, clean, slightly translucent, slightly attached to the stuffing, clean surface, evenly wrinkled, matte, slightly oily, light brown to brown in colour, single dark spice particles noticeable	characteristic for this product, edible casing, clean, tight adhering to the stuffing, clean, matte surface, evenly wrinkled, light brown in colour with numerous different grains visible
	60 days	characteristic for this product, edible casing, clean, dry, tightly attached to the stuffing, evenly wrinkled surface, matte, slightly greasy, light brown to brown in colour, with noticeable colour components, visible single dark spice particles	characteristic for this product, edible casing, matte to slightly shiny in places, tightly attached to the stuffing, surface clean, evenly wrinkled, matte, golden brown colour, visible numerous dark green small fragments of herbs and dark spice particles
	120 days	characteristic for this product, casing edible, clean, slightly translucent, tightly attached to the stuffing, surface clean, evenly wrinkled, matte, brown colour	characteristic for this product, edible casing, clean, tight adhering to the stuffing, matte surface, evenly wrinkled, light brown in colour with numerous different grains visible
Appearance at the cross-section	28 days	characteristic for this product, ingredients evenly distributed, pale pink meat with a cream shade to pink, cream to creamy yellow and orange-coloured additives	acceptable for this product, evenly distributed components visible on the cross-section, meat dried out of a light beige colour with a gently delicate light pink tinge, with numerous light cream and dark grains visible
	60 days	acceptable for this product, ingredients evenly distributed, fairly dried meat, cream-light brown in colour, with a slightly pink tinge in places, with visible single flecks of spices	acceptable for this product, ingredients evenly distributed, meat slightly dried, light pink in colour with a beige tinge, fat colour cream-coloured, colour seeds cream-beige to golden, visible quite numerous dark green small fragments of herbs and dark spice particles
	120 days	quite characteristic for this product, ingredients evenly distributed, meat slightly dried out, colour pink with a gently cream shade, groats creamy to pale yellow, seeds cream yellow and beige grey	characteristic for this product, evenly distributed components visible on the cross-section, meat gently dried out of a light beige colour to light pink tinge, with numerous light cream and dark grains visible
Structure and consistency	28 days	acceptable for this product, degree of fragmentation—ingredients finely ground, slightly tight, brittle, slightly dry, slightly soft, with perceptible additives	proper for this product, quite elastic, slightly brittle, gently hard, gently dry, with noticeable additives, degree of fragmentation—finely ground
	60 days	acceptable for this product, quite tight, brittle, dry, gently soft, with perceptible additives, degree of fragmentation—ingredients finely ground	proper for this product, quite elastic, slightly brittle, gently hard, gently dry, with noticeable additives, degree of fragmentation—finely ground
	120 days	proper for this product, degree of fragmentation—ingredients finely ground, tight, quite elastic, suitably soft, gently hard, gently brittle, with perceptible additives	quite proper for this product, quite elastic, slightly brittle, slightly hard, slightly dry, with noticeable additives, degree of fragmentation—finely ground

**Table 7:** The sensory descriptors of aroma and taste of the hybrid semi-dry ready-to-eat cabanossi sausages with groats compared with the hybrid semi-dry ready-to-eat cabanossi sausages with sunflower seeds after storage at 25 °C.

Descriptors	Time of Storage	Hybrid Semi-Dry Ready-to-Eat Cabanossi Sausages with Groats	Hybrid Semi-Dry Ready-to-Eat Cabanossi Sausages with Sunflower Seeds
Aroma	28 days	quite characteristic for this product and the ingredients used, slightly aromatic, slightly intense, well perceptible added spices	characteristic for this product and the ingredients used, medium aromatic, medium intense, good perceptible spices used, slightly smoky, without off-odours
	60 days	characteristic for this product and the ingredients used, slightly aromatic, slightly intense, well perceptible added spices, without off-odours	quite characteristic for this product and the ingredients used, medium aromatic, medium intense, slightly perceptible added spices, gently smoky, without off-odours
	120 days	characteristic for this product and the ingredients used, slightly aromatic, medium intense, well perceptible added spices, slightly smoky, without off-odours	characteristic for this product and the ingredients used, medium aromatic, medium intense, good perceptible spices used, slightly smoky, without off-odours
Taste	28 days	quite characteristic for this product and the ingredients used, medium expressive, clearly perceptible added spices and aromas, quite spicy, slightly salty, gently meaty, very gently sweet, well perceptible additives, without off-flavours	characteristic for this product and the ingredients used: expressive, slightly salty, slightly smoky, clearly perceptible spices and grains used, without off-flavours
	60 days	quite characteristic for this product and the ingredients used, medium expressive, mainly perceptible added spices and aromas, quite spicy, slightly salty, very gently sweet, without off-flavours	characteristic for this product and the ingredients used, medium expressive, clearly perceptible added spices and additives, slightly salty, without off-flavours
	120 days	characteristic for this product and the ingredients used, medium expressive, well perceptible added spices and additives, slightly salty, gently spicy, gently smoky, without off-flavours	characteristic for this product and the ingredients used, medium expressive, slightly salty, clearly perceptible spices and grains used, gently smoky, without off-flavours

## Data Availability

The original contributions presented in the study are included in the article, further inquiries can be directed to the corresponding author.
